# Locally Advanced Pancreatic Cancer: A Review of Local Ablative Therapies

**DOI:** 10.3390/cancers10010016

**Published:** 2018-01-10

**Authors:** Alette Ruarus, Laurien Vroomen, Robbert Puijk, Hester Scheffer, Martijn Meijerink

**Affiliations:** Department of Radiology and Nuclear Medicine, VU University Medical Center, 1081 HV Amsterdam, The Netherlands; la.vroomen@vumc.nl (L.V.); r.puijk@vumc.nl (R.P.); hj.scheffer@vumc.nl (H.S.); mr.meijerink@vumc.nl (M.M.)

**Keywords:** locally advanced pancreatic cancer (LAPC), pancreatic cancer, local ablative therapies

## Abstract

Pancreatic cancer is typically characterized by its aggressive tumor growth and dismal prognosis. Approximately 30% of patients with pancreatic cancer present with locally advanced disease, broadly defined as having a tumor-to-artery interface >180°, having an unreconstructable portal vein or superior mesenteric vein and no signs of metastatic disease. These patients are currently designated to palliative systemic chemotherapy, though median overall survival remains poor (approximately 11 months). Therefore, several innovative local therapies have been investigated as new treatment options for locally advanced pancreatic cancer (LAPC). This article provides an overview of available data with regard to morbidity and oncological outcome of novel local therapies for LAPC.

## 1. Introduction

Pancreatic adenocarcinoma is one of the most aggressive forms of cancer and is projected to arise as the second leading cause of cancer-related deaths in Europe and the United States by 2030 [1]. The prognosis has hardly improved over the past two decades and remains dismal, with an overall 5-year survival rate of approximately 8% [2,3]. Surgical resection is the only treatment option with the potential for long-term survival and cure. Even after potential curative resection, most patients will eventually have recurrent disease, resulting in a 5-year survival of only 20% [4]. Because early symptoms are often vague and mild, roughly 30% of patients with pancreatic cancer present with locally advanced pancreatic carcinoma (LAPC) and approximately 50% with metastatic disease (mPC) [5]. 

Systemic chemotherapy is considered the standard of care for patients with LAPC (AJCC stage III) and mPC (AJCC stage IV) [6]. The FOLFIRINOX regimen (combination chemotherapy using fluorouracil, leucovorin (folinic acid), irinotecan, and oxaliplatin) has emerged over the last years as a therapy that improves survival (median overall survival (OS) 11.1 months for FOLFIRINOX vs. 6.8 months for gemcitabine for mPC), at the cost of a greater concomitant toxicity [7]. International medical oncology guidelines extrapolate these results and hence recommend the use of FOLFIRINOX as the standard of care for mPC and for LAPC patients with a good performance status and no major comorbidities [4,8,9].

Considering the poor survival of LAPC patients, a lot of research from the last decade focused on combining systemic chemotherapy with local ablative therapies, such as radiofrequency ablation (RFA), microwave ablation (MWA), cryoablation, irreversible electroporation (IRE), stereotactic body radiation therapy (SBRT), iodine-125 seed implantation, high-intensity focused ultrasound (HIFU), and photodynamic therapy (PDT). The ablative techniques all share the mutual goal to achieve local tumor control, as this likely impacts quality-of-life and survival.

Thermal ablation techniques, such as RFA, MWA and cryoablation, attempt to destroy tumor tissue by increasing or decreasing temperatures sufficiently to induce cellular injury [10]. Complete and adequate destruction requires that the entire tumor plus the ablative margin to be subjected to cytotoxic temperatures. In RFA, a high-frequency alternating current runs through one or more electrodes, leading to tumor destruction by coagulation and protein denaturation [10]. In MWA, the oscillation of polar molecules produces frictional heating, ultimately generating tissue necrosis [11]. Cryoablation utilizes argon or helium gases to induce the rapid freezing and thawing of target tissue [12]. Cellular destruction by cryoablation is caused by direct physical damage during freezing, and by vascular-mediated cytotoxicity occurring as a result of progressive vasoconstriction, occlusion and endothelial damage, resulting in tissue ischemia [12]. Another thermal, but non-needle guided, ablative technique is HIFU. HIFU destroys tumor cells by raising local tissue temperatures as high as 65˚C using focused ultrasound energy from an extracorporeal source [13].

In contrast to conventional radiotherapy, which has shown conflicting results for LAPC [14,15,16], SBRT permits the precise application of high-dose radiation to a limited target volume, reducing the radiation dose adjacent to healthy tissue and subsequently minimizing toxicity. Another form of irradiation studied for pancreatic cancer is iodine-125 seed implantation (brachytherapy). Iodine-125 seed is an isotope which provides gamma radiation for a short distance, resulting in the death of targeted cells [17]. It has been suggested as a distinct treatment option or for use in combination with other ablative therapies.

In PDT, localized tissue necrosis is caused by the activation of a photosensitizer using light of a specific wavelength [18]. The photosensitizer causes a cytotoxic effect by the generation of reactive oxygen species (ROS), such as singlet oxygen and free radicals, that mediate cellular toxicity [18].

In the field of LAPC, IRE is the ‘new kid on the block’. In IRE, multiple needle electrodes are placed in and around the tumor, either percutaneously or during laparotomy. High-voltage electrical pulses are delivered between each needle electrode pair, creating nanopores that irreversibly damage the cell membrane, leading to apoptosis. Since the working mechanism is primarily non-thermal, there is little concurrent damage to connective tissues such as collagen and elastin, so the mechanical integrity of tissue or important structures, like blood vessels and bile ducts, is preserved [19].

In reversible electroporation, in contrast to IRE, a transient state of cellular permeabilization is created using electric pulses. This allows for extracellular agents to access the cells. Reversible electroporation combined with chemotherapeutic agents (ECT) may improve their uptake, in particular for drugs that are poorly, or not, permeant [20]. 

This article aims to give an extensive overview of the available literature regarding the safety and oncological outcome of these local innovative ablative therapies in the treatment of patients with LAPC.

## 2. Search

A search was performed in October 2017 in PubMed for studies published in the English language. Studies were eligible if they reported safety and/or oncological outcomes of at least ten patients treated for LAPC. Case reports, (systematic) reviews, animal or experimental/preclinical studies, and studies reporting multiple tumor histologies or stages were excluded. Abstracts were merely included when their methodology and all relevant data could be adequately extracted.

## 3. Radiofrequency Ablation (RFA)

Six studies included at least ten patients; four prospective [21,22,23,24] and two retrospective [25,26]. The RFA procedures were performed during open laparotomy in five studies and using a percutaneous approach in one. All six studies were performed at the University of Verona Hospital Trust. Given the intersecting date ranges, the results likely stem from a (partially) overlapping patient cohort [21,22,23,25,26]. Patients in the study from D’Onofrio et al. were treated with percutaneous RFA [24]. Four studies reported on OS, ranging between 19.0 and 25.6 months [21,22,25,26]. The study from Cantore et al. compared upfront RFA plus palliative bypass surgery to RFA as a second-line treatment option after chemo(radio)therapy. The median OS from date of diagnosis was significantly higher for patients receiving RFA as a second-line treatment compared to upfront RFA (25.6 months vs. 14.7 months; *p* = 0.004). However, as the number of patients that did not qualify for RFA after chemo(radio)therapy is unclear, an important selection bias remains. Morbidity varied between 0 and 28% and 30-day mortality between 0 and 3%, which was caused by hepatic failure [21,22,23], sepsis following a duodenal perforation [21], severe acute pancreatitis [22], and duodenal hemorrhage [22]. The most common (serious) adverse events included pancreatic fistula [21,22,23,25], acute pancreatitis [21,22,23], portal vein thrombosis [21,22,23], duodenal injury [21,22,23,25], biliary injury [21,22,23], gastric ulcer or fistula [22,23,25], haemoperitoneum [21,22,23], and liver failure [21,22,23]. An overview of the literature on RFA is given in Table 1.

## 4. Microwave Ablation (MWA)

Currently available data on microwave ablation for LAPC is limited. Lygidakis et al. studied the feasibility, safety, and efficacy of MWA in 15 patients with histologically proven LAPC [27]. In all patients, partial necrosis was achieved whilst no major procedure-related morbidity or mortality occurred. Carrafiello and colleagues retrospectively reviewed ten patients treated with percutaneous (*n* = 5) or laparotomic (*n* = 5) MWA [11]. The 9-month and 1-year local tumor progression rates per new response evaluation criteria in solid tumours (RECIST 1.1) were 37.5% (3/8) and 62.5% (5/8), respectively [28]. In 20% of the patients, minor complications were registered. Two grade 3 or more complications were registered: pancreatitis (grade 3; *n* = 1) and pseudoaneurysm of the gastroduodenal artery (grade 4; *n* = 1). 

## 5. Cryoablation 

Two studies compared cryoablation plus palliative bypass surgery (PBC group) to bypass surgery alone (PB group) [29,30]. Although both studies found tumor mass shrinkage in the PBC group, OS was not significantly different from the patients in the PB group (350 days versus 257 days, *p* = 0.124; 5 months versus 4 months, *p* > 0.05). The postoperative complication rate in the study from Li et al. was not significantly different between the two groups, except for delayed gastric emptying, which was higher in the PBC group (35.7% in PBC group vs. 5.3% in PB group) [29]. Main postoperative complications included pancreatic or biliary leakage, GI bleeding or obstruction, delayed gastric emptying, infection or intra-abdominal bleeding [29,30].

## 6. High-Intensity Focused Ultrasound (HIFU)

Ten studies were identified that reported on pain relief, morbidity or oncologic outcome after HIFU. Six studies were prospective [31,32,33,34,35,36] and four were retrospective [37,38,39,40]. Six studies reported on OS, which ranged between 6.0 and 14.0 months [31,32,35,37,39,40]. In the retrospective analysis from Ning et al., no significant survival benefit was found for patients treated with HIFU (median OS 8.3 months) compared to patients who did not receive HIFU treatment (median OS 7.3 months; *p* = 0.783) [37]. Gao et al. compared patients treated with HIFU alone to patients treated with HIFU and concurrent chemotherapy (gemcitabine 1000 mg/m^2^ over 30 min, weekly for 3 weeks every 28 days) [31]. The median OS was significantly longer for patients treated with both HIFU and chemotherapy (median OS 12.0 months versus 8.0 months; *p* < 0.05) [31]. Morbidity was relatively low, varying between 0–23.2% [31,32,33,35,37,39,41]. Increase of serum amylase was commonly seen after HIFU [31,34,36,37,40,41]. Other regularly encountered complications included skin burns or subcutaneous fat sclerosis [34,39,40,42], GI dysfunction (e.g., nausea, vomiting, loss of appetite) [31,34,36,37,41], GI ulcer or bleeding [32,34,37,41,42], abdominal pain [37,38,40,41], (mild) fever [34,36,37,40], pancreatic fistula or pseudocyst [33,34,39,40] and pancreatitis [33,34,36,42]. Eight studies reported on pain relief after HIFU, which is a major focus of HIFU treatment [31,32,33,34,35,36,38,39]. Pain relief was achieved in 78.6% to 87.5% of patients, either complete or partial (i.e., numerical (pain) rating scale (NRS) decrease by 2 or more) [31,35,36,39]. A summary of literature on HIFU is given in Table 2.

## 7. Stereotactic Body Radiotherapy (SBRT)

Nineteen single arm studies have been published regarding SBRT for LAPC; 9 prospective [43,44,45,46,47,48,49,50,51] and 10 retrospective [52,53,54,55,56,57,58,59,60,61]. Some studies have overlapping study populations, as the retrospective analysis of Alagappan et al. [52] included 72 patients that were already included in other reports [46,47,49,50]. Furthermore, the study from Mellon et al. [57] updated the outcomes and toxicity using induction chemotherapy and SBRT from an earlier published report by Chuong et al. [54]. The treatment modalities included SBRT with a linear accelerator [43,46,50,51,52,54,57,59] or CyberKnife [45,47,48,49,52,53,55,56,59,60,61]. In two studies, the treatment modality was not specified [44,58]. Median radiation doses varied between 20 Gy, delivered in one fraction, and 45 Gy, in six fractions. 

All studies provided data on OS (range: 10–20 months from diagnosis [43,44,46,47,48,49,50,52,53,54,55,56,57,58,61] and 6.2–12.5 months from SBRT [51,53,59,60]) after a median follow-up varying between 5 and 24 months. Local control varied between 40 and 89% [43,44,45,46,47,48,49,50,51,52,53,54,55,56,57,59]. Ten studies reported the number of patients who were eligible for resection after SBRT, which ranged from 0 to 20.3% [43,45,46,48,49,50,53,54,57,58]. An overview of the efficacy of SBRT for LAPC is given in Table 3. Complications from SBRT were divided into acute (within 3 months after SBRT) and late complications. Acute grade 3 or higher toxicity ranged between 0 and 28.4%, whilst late grade 3 or higher toxicity varied between 0 and 13% [43,45,46,47,48,49,50,51,53,54,55,56,57,58,59,60,61]. Frequently encountered mild to moderate complications include fatigue, abdominal pain and nausea. Other common complications were mostly GI-related, such as ulceration [46,49,50,53,57,58], gastritis or enteritis [43,46,49,58], duodenal stricture [49,53,60,61], bleeding from the gastro-intestinal tract [46,54,55,56,57,58], or anorexia [46,54,57,61]. 

## 8. Iodine-125 Seed Implantation

Two studies from Wang et al. treated a total of 28 patients (who were considered unresectable) during laparotomy with ^125^I seed implantation [62,63]. Median irradiation dose was 120 Gy (range 60–163 Gy). Seven patients received an additional 35–50 Gy external beam radiotherapy (EBRT) and ten patients received 2–10 cycles of adjuvant chemotherapy. The overall local control rate was 87.5% (*n* = 24), median OS 10.1 months. In the majority of patient pain relief was classified as good or medium (94.1%). Adverse events included a chylous fistula (*n* = 1), gastric ulcer (*n* = 1), radiation enteritis (*n* = 2), and transient fever (*n* = 10). In two patients, seeds migrated to the liver, however without any side effects. 

Xu et al. investigated the combination of ^125^I seed implantation with cryosurgery in 49 patients with LAPC, of which 12 patients had hepatic metastases [64,65]. Seeds were implanted during cryosurgery in 35 patients, or 3–9 days after cryosurgery in 14 patients. Twenty patients received additional (1–4 cycles) chemotherapy. After a median follow-up (FU) of 18 months, the median survival was 16.2 months. The majority of patients experienced abdominal pain (*n* = 34) and/or fever (*n* = 26). Other adverse events included acute pancreatitis (*n* = 6), increased amylase levels (*n* = 25), abdominal bleeding (*n* = 3), pulmonary infection (*n* = 3), myocardial infarction (*n* = 1) and cerebral infarction (*n* = 1). Iodine-125 seed implantation has also been investigated in combination with RFA by Zou et al. [66]. Twenty-four patients with stage III pancreatic cancer, identified during laparotomy, were treated with intraoperative RFA plus seed implantation. Pain scores decreased significantly after the operation (*p* < 0.05). The median OS was 19 months. One patient experienced acute pancreatitis, which was related to the RFA procedure. 

## 9. Irreversible Electroporation (IRE)

Fourteen single arm studies were published regarding IRE for LAPC, either as margin accentuation [67,68,69,70] or as primary tumor treatment [71,72,73,74,75,76,77,78,79,80]. Patients were treated percutaneously [69,71,72,73,74,75,77,80], laparoscopically [70], or during laparotomy [68,69,70,72,73,76,78,79]. After a median follow-up of 1–29 months, the median OS from diagnosis ranged between 15.3 and 27.0 months [68,70,74,75,76,77,78], whilst the median OS from IRE varied between 7.0 months and 14.2 months [67,73,74,75,77,81]. In the study from Martin et al., the difference in OS between patients treated with IRE for primary tumor control (median OS: 23.2 months) was compared to IRE as margin accentuation after resection (median OS: 28.3 months). However, this was not found to be statistically significant (*p* > 0.05) [68]. Three studies reported on the ability of resection after IRE, ranging between 6.0 and 10.3% [74,75,81]. Local progression was specified in 9 studies, varying between 0 and 27.8% [67,68,69,70,72,75,80,81]. Mansson et al. found a local failure rate of 58.3%, however, this number may have been overestimated since the authors rated every ablation zone growth as local progression [74]. Table 4 gives an overview of the efficacy of IRE for LAPC.

Major complications (grade 3 or higher) varied between none to 30% [67,71,74,75,80], whilst overall morbidity ranged between 10 and 57% [68,69,72,73,74,76,77,78,79]. Most frequently encountered (major) complications were abdominal pain [75,77]; GI-related adverse events such as nausea and anorexia [67,68,70,72,77], delayed gastric emptying or ileus [67,69,70,72,74,77,78,79], and (perforated) ulcers with or without GI bleeding [67,74,77,78,79]; portal vein thrombosis [67,68,69,70,72,74,75,78,79]; biliary stricture; leakage or cholangitis [67,68,69,70,72,73,77,78]; pancreatic leakage; fistula or pancreatitis [70,73,74,75,76,77,78,79]; abscesses [67,73,76,77]; and ascites [67,68,69,70,72].

Two studies did not report mortality rates [71,72], whilst four studies did not have any 90-day mortality [73,76,77,80]. In the other studies, the mortality varied between 1 to 3 patients [67,68,69,70,73,74,75,78,79]. Mortality was caused by tumor progression [75,79], liver failure [68,78], hemorrhage from a GI ulcer [68,78], portal vein thrombosis [67,69,70], duodenal and bile duct necrosis [67], multisystem organ failure [67], cardiopulmonary arrest [67], pneumonia [74], and pulmonary embolism [68].

## 10. Photodynamic Therapy (PDT)

One study, performed by Huggett et al., was published investigating PDT for patients with LAPC [82]. An earlier study from the same group studied the photosensitizer meso-tetrahydroxyphenyl chlorin (mTHPC), however, this study also included patients with stage 1 and 2 pancreatic cancer, whilst data for stage III pancreatic cancer could not be extracted separately [83]. The study from Huggett et al. used the photosensitizer verteporfin in 15 patients treated for LAPC, with a median tumor size of 4.0 cm [82]. One and three months after IRE, 11 and 6, respectively, out of 13 patients had stable disease. The median OS after PDT was 8.8 months and from diagnosis 15.5 months. Adverse events included mild to moderate pain (*n* = 3), transient increase in amylase levels (*n* = 1), mild diarrhea (*n* = 1), persistent steatorrhea (*n* = 1), and subclinical inflammatory changes on computed tomography (CT) (*n* = 2). 

## 11. Electrochemotherapy (ECT)

Experience with ECT for LAPC is limited. Granata et al. investigated the safety and feasibility of ECT in 13 patients with LAPC [84]. No electrochemotherapy-related serious adverse events occurred. A transient, self-limiting supraventricular arrhythmia was detected in one patient. In four patients, delayed gastric emptying occurred, however without clinical significant symptoms. Other complications included: pleural effusion, ascites, and splenic infarction without thrombosis of the splenic vessels. The same research group also published an article regarding early radiological assessment of LAPC treated with ECT, elaborating the cohort to 19 patients [84,85]. One patient died within 48 h after treatment with ECT because of a complication, which has not been discussed in detail. No significant reduction of largest diameter by CT scan and magnetic resonance imaging (MRI) was observed. According to RECIST criteria, all patients showed stable disease using MRI, while on CT imaging one patient showed progressive disease. According to Choi criteria, all patients were considered in partial response. Using functional MR derived parameters, a significant reduction of viable tumor tissue was observed. 

## 12. Discussion

This study presents an overview of the available literature on local ablative therapies for LAPC. The most data was available for RFA, SBRT, IRE and HIFU. The main shortcoming affecting all techniques is the lack of randomized controlled trials determining the (additional) value of local ablative technique above systemic palliative chemotherapy alone. The comparative analysis of treatment options is also hampered by the fact that until recently there was no globally accepted standard of care for LAPC [86] Potential selection biases, such as the lack of consensus regarding resectability criteria and the heterogeneous use of (neo-)adjuvant chemo(radio)therapy plus consequential timing of the ablative procedure, further impede a reliable assessment [21,86,87,88]. Since protracted courses of neo-adjuvant therapy will exclude patients with early disease progression from receiving ablative therapy, the OS for the ablated group will likely increase given the biologically favorable nature of the tumor. Nonetheless the reported OS, especially after SBRT and IRE, remains promising. Besides the goal to achieve local tumor control, downstaging to resectable disease has also been reported after local ablative therapies as SBRT and IRE (see Table 3 and Table 4). Although the number of patients that were able to undergo resection is low, local ablative therapies can create curative potential for patients who were actually designated to palliative therapy only.

**Figure 1:**
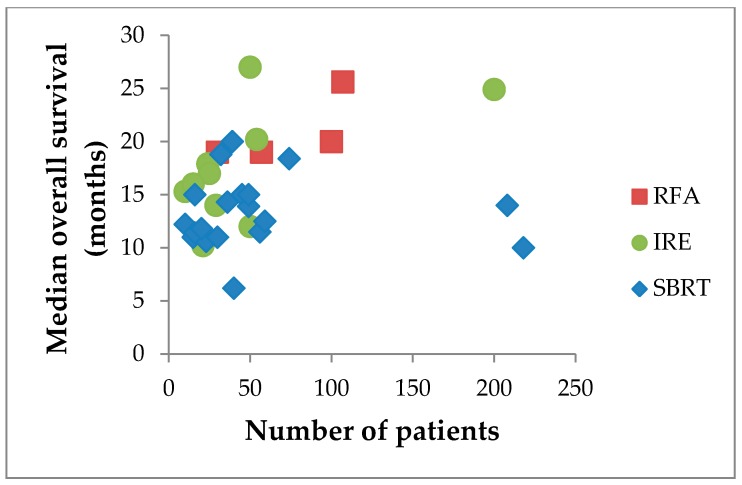
Median overall survival in months after RFA*, SBRT and IRE per number of patients per study. * All procedures in all studies performed at one single center: the University of Verona Hospital Trust; given the overlapping date ranges patient data are likely (partially) re-reported.

All local therapies described in this article have their own distinct advantages and disadvantages (Table 5). Although the survival benefit of adding traditional fractionated external-beam radiotherapy to chemotherapy regimens remains controversial [14,15,16], SBRT allows for limited fractions, decreased toxicity, and more promising survival outcomes (median OS reaching 20 months; Figure 1) [56,89,90]. Furthermore, patients can be treated in the outpatient setting because the technique is minimally invasive, with the exception of the need to implant fiducials [89]. The latter may be overcome by MRI-guided radiation therapy [91]. This technique continuously images soft-tissue during radiation treatment, allowing for accurate alignment of the tumor to the treatment beams, and does not require fiducials [91]. Physicians should be aware of the risk of late complications (i.e., >3 months after SBRT) and the inability of retreatment in the case of local failure. The literature on SBRT is heterogeneous with regards to delivered radiation doses and fractions, which confounds the comparison of SBRT to other local ablative therapies [90].

Similar to SBRT, HIFU does not require needle placement. Although the number of articles on HIFU for LAPC is relatively high, the number of patients per series was limited and the majority focused on pain relief without reporting oncological outcomes [92]. Pain can effectively be relieved by HIFU in 78.6–87.5% of patients, albeit at the cost of major skin burns (grade 2 or 3) and/or subcutaneous fat sclerosis, [31,35,36,39].

Due to the anatomical location of pancreatic cancer, RFA in the pancreas is associated with a high morbidity and mortality, since heating can seriously damage critical blood vessels, bile ducts and gastro-intestinal structures [93]. For this reason RFA has been largely abandoned, with the exception of one group who advocates the use of a large safety margin to these critical structures. Hence the primary aim of the technique is cytoreduction [22,87]. The continuous cooling caused by nearby blood vessels may further negatively impact the effect of RFA (heat-sink effect) [93]. On the other hand, RFA is relatively easily applicable, has a superior availability, and low costs. Pancreatic RFA is mostly performed during open laparotomy, which has the advantage of the exploration of the peritoneal cavity to identify unsuspected disease and therefore withhold patients from unnecessary treatment with RFA. Although the percutaneous approach may be more suitable to palliate patients, only one study investigated this approach [24].

Similar to thermal ablation, IRE can also be performed during open laparotomy or percutaneously. IRE has the theoretical advantage that the goal is to radically ablate the entire macroscopically visible tumor and hence achieve local tumor control. Since the working mechanism of IRE is based on the destruction of cellular membranes, whilst preserving the extracellular matrix, blood vessels and bile ducts remain intact [93]. Moreover, since the working mechanism is not based on thermal energy, IRE is not affected by the heat-sink effect [93]. These two characteristics make the use of pulsed electrical fields a promising technique for LAPC. These statements seem to be supported by the OS found in literature (range 15.3–27.0 months; Figure 1) [94]. However, IRE is known to have a high learning curve and literature is heterogeneous with regards to the electrical settings used for the procedure [95]. This latter problem is currently being addressed by an international Delphi consensus study, aiming for a uniform applied protocol. The morbidity and mortality rates reported in the more recent prospective series seem to be higher than those from earlier retrospective reports, especially for the open approach [77].

The other techniques discussed in this review, i.e., PDT, MWA, cryoablation, ECT, and iodine-125 seed implantation, all proved feasible in the treatment of LAPC patients. However, the data is too limited to draw any hard conclusions with regards to safety and efficacy.

## 13. Future Perspectives

As stated before, the main shortcoming of all local therapies is the lack of randomized controlled trials, comparing the additional value of local therapy over systemic chemotherapy. The currently ongoing CROSSFIRE-trial (ClinicalTrials.gov number NCT02791503), is a randomized controlled phase III trial comparing the outcome of FOLFIRINOX plus IRE with FOLFIRINOX plus MR-guided SBRT on OS for patients with LAPC. The PELICAN-trial (Dutch Trial Register number NTR5517), is another ongoing randomized controlled trial that compares the outcome on OS for patients with LAPC treated with FOLFIRINOX or gemcitabine plus RFA to treatment with chemotherapy only. The randomized phase III trial, organized by the Stanford University, aims to determine the additional value of SBRT over FOLFIRINOX alone (ClinicalTrials.gov number NCT01926197). These trials will hopefully further define the exact role of local therapies for patients with LAPC.

The current focus of several studies is the immunogenic potential of local ablative therapies. After the destruction of the tumor with a local ablative technique, antigen presenting cells (APCs) infiltrate the ablation zone [98]. These APCs then activate the immune system, specifically helper and cytotoxic T cells [98]. This may induce a so-called ‘abscopal effect’, referring to the phenomenon where localized treatment of the primary tumor induces a forceful immune response, targeting occult distant micrometastases, potentially prolonging tumor control and survival [99]. Besides the activation of the immune response, small studies also have shown a decrease of immunosuppressive cells after treatment with local ablative techniques [96]. Combining local ablative techniques with immunotherapy may potentially boost the immune system to suppress pancreatic cancer [97]. Another direction for future studies on LAPC is the correlation of treatment effectiveness or survival with genetic alterations. There are four commonly known mutated genes in pancreatic cancer: KRAS, CDKN2A, TP53, and SMAD4 [3]. Studies of precursor lesions found KRAS mutations to be one of the earliest alterations in pancreatic tumorigenesis, along with CDKN2A [3,100,101,102]. In contrast, the inactivation of SMAD4 and TP53 were found in advanced pancreatic intraepithelial neoplasias grade 3 and invasive carcinomas [102]. A recent study from Paiella et al. showed patients with pancreatic cancer with a SMAD4 loss had a poorer prognosis after RFA [26]. In the future, it may be possible to identify subgroups of patients who will and will not benefit from local therapies. 

Also currently investigated, though not addressed in this paper, are oncolytic viruses [103,104]. Viruses are designed with biological specificity to infect cancerous cells preferentially [104]. The direct working mechanism is overwhelming viral infection and lysis, which releases additional viral particles to infect neighboring cells and distant metastases [104]. Viral infections can also activate the immune system and aid the immune system to recognize and attack malignancies [104]. For instance, in the randomized phase II trial performed by Noonan et al., pelareorep (reolysin) was added to treatment with chemotherapy (carboplatin and paclitaxel) [103]. Pelareorep has cytotoxic effects on malignant cells with an activated RAS signaling pathway, due to mutations in the RAS proto-oncogene [103]. No survival benefit was found for patients treated with pelareorep, which could be due to a neutralized effect in the context of other (unknown) mutations. Although patients receiving pelareorep did not experience a survival benefit, a number of immune biomarkers were found that were associated with an improved disease control rate or progression free survival [103].

## 14. Conclusions 

In conclusion, this review gives an overview of the currently available ablative techniques for the treatment of patients with LAPC, with their distinct advantages and disadvantages. Although the safety profile is generally defined as good, major adverse events can occur and even mortality has been reported for all techniques. In the absence of randomized controlled trials, high-quality evidence for any survival benefit over chemotherapy alone is lacking. Only one series, comparing IRE plus chemotherapy to chemotherapy alone, has used case matching and multivariate analysis to compare two historical cohorts [70]. Although OS was superior in the IRE group, several confounders remain. Nonetheless, the promising survival, especially for studies employing SBRT and IRE, warrant the setup of randomized controlled phase II and III trials to compare the available local ablative therapies and to assess the additive value of local therapy over chemotherapy alone.

## Figures and Tables

**Table 1:** Safety and efficacy of radiofrequency ablation (RFA) for locally advanced pancreatic cancer (LAPC).

Reference	Design	# pts	Age, yrs	Size, mm	Morbidity	30-day Mortality	Median FU	Median OS
Cantore [21] **	Prospective	107	N.S.	N.S.	28.0% (*n* = 30)	1.9% (*n* = 2)	N.R.	25.6 months
D’Onofrio [24] **	Prospective	18	mean 62.4	mean 48.1 (25–86)	0%	0%	N.R.	N.R.
Frigerio [25] **	Retrospective	57	med 63	N.R.	14% (*n* = 8)	0%	N.R.	19 months
Girelli [23] **	Prospective	50	med 64.5	med 40 (IQR 30–50)	24% (*n* = 12)	2% (*n* = 1)	8 months	N.R.
Girelli [22] **	Prospective	100	mean 64	med 36 (IQR 30–45)	26% (*n* = 26)	3% (*n* = 3)	12 months	20 months
Paiella [26] **	Retrospective	30	N.R.	N.R.	N.R.	0%	15 months	19 months *

pts = patients; FU = follow-up; OS = overall survival; med = median; IQR = interquartile range; N.S. = not specified (per group); N.R. = not reported; * median disease specific survival. ** All procedures in all studies were performed at one single center: the University of Verona Hospital Trust; given the overlapping date ranges patient data are likely (partially) re-reported.

**Table 2:** Safety and efficacy of high-intensity focused ultrasound (HIFU) for locally advanced pancreatic cancer (LAPC).

Reference	# pts	Age, yrs	RECIST	Median OS	Morbidity	Pain Relief
Gao [31]	39	med 58 (42–79)	CR 0 PR 5 (12.8%) SD 25 (64.1%) PD 9 (23.1%)	11.0 months	12.8%	Total 31 (79.5%) Complete 9 (23.1%) Partial 22 (56.4%) †
Li [32]	16	mean 62 (49–72)	CR 0% * PR 43.7% SD 25% PD 31.3%	14.0 months (from treatment)	12.5%	Mean pre-VAS 5.1 Mean post-VAS 3.3 Median PRT 5.6 months
Ning [37]	100	N.S.	N.R.	8.3 months	23.2%	N.R.
Shi [38]	71	N.R.	N.R.	N.R.	N.R.	Pre-HIFU 70.42% painless Post-HIFU 92.96% painless
Sofuni [33]	30 16 III	N.S.	N.S.	N.R.	10%	66.7% (N.S.) ‡
Sung [34]	46 18 III	N.S.	N.R.	N.S.	N.R.	Pre-VAS 4.9 Post-VAS 2.1 *p* < 0.001 (N.S.)
Wang [35]	40 13 III	N.S.	N.S.	10 months	0%	Total 35 (87.5%) (N.S.) Complete 9 (22.5%) Partial 26 (65%) Median PRT 10 weeks
Xiong [39]	89 39 III	N.S.	N.S.	11.2 months	11.2%	Total 54 (80.6%) (N.S.) Complete 21 (31.3%) Partial 33 (49.3%) †
Zhao H. [36]	39 31 III	N.S.	N.S.	N.S.	N.R.	Total 22 (78.6%) (N.S.) Complete 9 (32.1%) Partial 13 (46.4%) †
Zhao J. [40]	38	med 75 (62–80)	N.R.	6.0 months vs. 10.3 months ~	N.R.	N.R.

pts = patients; N.S. = not specified (per stage); N.R. = not reported; III = stage 3 pancreatic cancer; VAS = visual analog scale; median PRT = median duration of pain relief time; RECIST 1.1 = new response evaluation criteria in solid tumours [28] * at 6 months after treatment; † NRS decrease by 2 or more; ‡ more than 30% improvement; ~ traditional HIFU 6 months vs. low power HIFU 10.3 months; *p* = 0.018.

**Table 3:** Efficacy of stereotactic body radiotherapy (SBRT) for locally advanced pancreatic cancer (LAPC).

Reference	Design	# pts	Age, yrs	Median Dose	Fractions	Median FU	Local Control	Median OS	Downstage
Alagappan [52]	Retrospective	208 *	med 75.2 (IQR 65.9–86.1)	25 Gy (103 pts) 33 Gy (105 pts)	1 5	7.5 months	87%	14.0 months (OSd)	N.R.
Chang [53]	Retrospective	77 56 III	N.S.	25 Gy (61 pts) 25 Gy + EBRT	1 1 + 25	6 months	87% (N.S.)	6.7 months (OSt) ‡ 11.5 months (OSd) ‡	1 pt (N.S.)
Chuong [54]	Retrospective	73 16 III	N.S.	30 Gy	5	7.8 months	1-year LC = 81% (N.S.)	15 months (OSd) ‡	0
Comito [43]	Prospective	45	mean 68 (40–87)	45 Gy	6	13.5 months	89%	15 months (OSd)	3 pts
Dholakia [44]	Prospective	32	N.R.	33 Gy	5	13.4 months	72%	18.8 months (OSd)	N.R.
Gurka [45]	Prospective	10	mean 62.5 (50–79)	25 Gy	5	N.R.	40%	12.2 months (N.S.)	0
Herman [46]	Prospective	49	med 67 (35–87)	33 Gy	5	13.9 months	max. 78%	13.9 months (OSd)	5 pts
Koong [47]	Prospective	15	med 62 (43–82)	20 Gy	1	5 months	max. 80%	11 months (OSd)	N.R.
Mahadevan 2011 [56]	Retrospective	39	med 67 (44–88)	24.92 Gy	3	21 months	85%	20 months (OSd)	N.R.
Mahadevan 2010 [55]	Retrospective	36	med 65 (43–88)	29.33 Gy	3	24 months	78%	14.3 months (OSd)	N.R.
Mellon [57]	Retrospective	159 49 III	med 67.2 (47–85)	40 Gy	5	14.0 months	1-year LC = 78% (N.S.)	15.0 months (OSd) ‡	5 pts
Moningi [58]	Retrospective	88 74 III	N.S.	33 Gy	5	14.6 months	N.R.	18.4 months (OSd) ‡	15 pts
Polistina [48]	Prospective	23	med 68 (44–75)	30 Gy	3	9 months	82%	10.6 months (OSd)	2 pts
Rwigema [59]	Retrospective	71 40 III	N.S.	24 Gy	1–3	6.0 months	53%	6.2 months (OSt) ‡	N.R.
Schellenberg 2008 [49]	Prospective	16	med 69 (39–87)	25 Gy	1	9.1 months	81%	11.4 months (OSd)	0
Schellenberg 2011 [50]	Prospective	20	med 63 (45–85)	25 Gy	1	N.R.	75%	11.8 months (OSd)	0
Song [60]	Retrospective	59	med 62 (28–86)	45 Gy	5 (3–8)	10.9 months	N.R.	12.5 months (OSt)	N.R.
Tozzi [51]	Prospective	30	mean 67 (43–87)	45 Gy	6	11 months	86%	11 months (OSt)	N.R.
Zhu [61]	Retrospective	417 218 III	N.S.	30–46.8 Gy	5–8	11 months	N.R.	10.0 months (OSd) ‡	N.R.

pts = patients; OSd = median overall survival from diagnosis; OSt = median overall survival from treatment (SBRT); III = number of patients with stage 3 pancreatic cancer (LAPC); N.R. = not reported; IQR = interquartile range; N.S. = not specified per stage; LC = local control; EBRT = external-beam radiotherapy; * 12 patients had metastatic disease; ‡ overall survival of patients with LAPC only.

**Table 4:** Efficacy of irreversible electroporation (IRE) for locally advanced pancreatic cancer (LAPC).

Reference	# pts	Age, yrs	Size, mm	Approach	Treatment	Median FU	Median OS	Local Failure	Down-Stage	Mortality
Belfiore [71]	29	med 68.5 (55–81)	N.R.	Perc	Local	29 months	14 months (OSt)	3%	N = 3	N.R.
Dunki–Jacobs [72]	65	N.R.	med 35	Perc 12 Open 53	Local	23 months	N.R.	26%	N.R.	N.R.
Kluger [67]	50	med 66.5 (IQR 60.2–72.0)	med 30 (IQR 17–50)	N.R.	Margin 24 Local 29	8.69 months	12.03 months (OSt)	11%	N.R.	6% (*n* = 3) *
Lambert [73]	21	68.2	39 (21–65)	Perc 2 Open 19	Local	N.R.	10.2 months (OSt)	N.R.	N.R.	0
Mansson [74]	24	med 65 (42–77)	med 35 (15–45)	Perc	Local	N.R.	17.9 months (OSd) 7.0 months (OSt)	58.3%	N = 2	4% (*n* = 1)
Martin 2012 [69]	27	med 61 (45–82)	med 30	Perc 1 Open 26	Margin 8 Local 19	90 days	N.R.	0%	N.R.	4% (*n* = 1)
Martin 2013 [70]	54	med 61 (45–80)	N.R.	Open 52 Lap 2	Margin 19 Local 35	15 months	20.2 months (OSd)	27.8%	N.R.	2% (*n* = 1)
Martin 2015 [68]	200	med 62 (27–88)	med 28	Open	Margin 50 Local 150	29 months	24.9 months (OSd)	6%	N.R.	2% (*n* = 3)
Narayanan [75]	50	med 62.5 (46–91)	mean 32 (15–80)	Perc	Local	N.R.	27.0 months (OSd) 14.2 months (OSt)	18%	N = 3	6% (*n* = 3)
Paiella [76]	10	med 66	med 30 (25–39)	Open	Local	7.6 months	15.3 months (OSd)	N.R.	N.R.	0
Scheffer [77]	25	med 61 (41–78)	med 40 (33–50)	Perc	Local	12 months	17 months (OSd) 11 months (OSt)	N.R.	N.R.	0
Vogel [78]	15	N.R.	N.R.	Open	Local	24 months	16 months (OSd)	N.R.	N.R.	13% (*n* = 2)
Yan [79]	25	med 58 (49–80)	med 42 (28–49)	Open	Local	N.R.	N.R.	N.R.	N.R.	4% (*n* = 1)
Zhang [80]	21	N.R.	med 35 (20–67)	Perc	Local	1 month	N.R.	0	N.R.	0

pts = patients; perc = percutaneous; lap = laparoscopic; OSd = overall survival from diagnosis; OSt = overall survival from treatment; N.R. = not reported; med = median; IQR = interquartile range; *3 deaths were deemed IRE related.

**Table 5:** Overview of specific advantages and disadvantages for various techniques.

Technique	Advantage	Disadvantage
RFA	Easily applicable; superior availability; low costs. Open approach allows for exploration of peritoneal cavity; percutaneous approach seems less invasive, however limited data (one study) for LAPC. Indication of RFA-based immunomodulation: general activation of adaptive immune response along with a decrease of immunosuppression [96].	Tumor debulking, since a safety margin is required to prevent thermal damage to critical structures such as large blood vessels and bile ducts. All available literature from one single center. Heat-sink effect, decreasing treatment efficacy of tumors surrounding large vessels. 30-day mortality (0–3%); relatively high complication rate: 0–28%.
MWA		Limited data for pancreatic cancer
Cryoablation	Presumed abscopal effect, especially when combined with immunotherapy [97].	Cryoshock syndrome. Hemorrhage induced by ice-ball cracking. Probe-size demands open approach. No survival benefit for cryoablation with palliative bypass surgery versus bypass surgery alone.
HIFU	No needles required. Effective technique for pain relief.	Limited survival data. Complication rate: 0–23.2%. Risk of second and third degree skin burns and subcutaneous fat sclerosis.
SBRT	Noninvasive, except for the implantation of the fiducials (though, very low complication rate). Treatment in the outpatient setting.	No uniform data with regard to radiation doses used, making comparisons difficult. Retreatment often impossible. Lower dose at the border of the tumor due to organs at risk (OARs). Risk of late complications (i.e., >3 months after SBRT): 0–13% (≥grade 3); acute complication rate: 0–28.4% (≥grade 3).
Iodine-125 seeds		Limited data for pancreatic cancer. Implantation demands open approach.
IRE	Deployable as primary tumor control or margin accentuation after resection. Treatment is repeatable. Preservation of critical structures, such as biliary ducts and large blood vessels. Not susceptible to heat-sink effect. Open approach allows for exploration of peritoneal cavity; percutaneous approach is less invasive.	No uniform protocol. High learning curve. 90-day mortality (0–13%); relatively high complication rate: 0–30% (≥grade 3).
PDT	Preservation of connective tissues, maintaining the mechanical integrity of critical structures, such as intestines and blood vessels.	Limited data for pancreatic cancer.
